# Anti-Vivisection Hospital

**Published:** 1910-08-20

**Authors:** George W. F. Robbins

**Affiliations:** Secretary


### Editor's Letter-Box.

### ANTI-VIVISECTION HOSPITAL.

*To the Editor of* The Hospital.

Sir,—My attention has just been called to certain statements made by Sir William Church, K.C.B., at a meeting of the Metropolitan Hospital Sunday Fund, held at the Mansion House on Wednesday, July 27 last, as quoted in your issue of July 30 :—

1. "The circular was sent to the Battersea General Hospital, but no reply had been received therefrom."
2. "That hospital had declined to give the Council any information about its funds separately from the Sunday collections."
3. "No reply had been received to a second letter."

The answer to 1 and 3 is, that my last two letters to the Secretary of the Fund were under dates July 13 and July 18—both some days before the meeting on July 27.

In regard to No. 2, the sentence seems hardly intelligible. But I may say that the hospital has not declined to give any such information, or any other information in respect of any audited accounts.

Yours faithfully,

George W. F. Robbins, Secretary.

